# Metabolomics Study of Flavonoids of *Taxillus* *chinensis* on Different Hosts Using UPLC-ESI-MS/MS

**DOI:** 10.3390/molecules26247681

**Published:** 2021-12-19

**Authors:** Li Li, Jianbei Teng, Yilin Zhu, Fengfeng Xie, Jing Hou, Yuan Ling, Hua Zhu

**Affiliations:** 1 College of Pharmacy, Chengdu University of TCM, Chengdu 611137, China; lil2014@gxtcmu.edu.cn; 2 College of Pharmacy, Guangxi University of Chinese Medicine, Nanning 530200, China; Tengjianbei@163.com (J.T.); nnzhyl@163.com (Y.Z.); 15177143553@163.com (F.X.); hj18878793603@163.com (J.H.); l1220175714@163.com (Y.L.)

**Keywords:** *Taxillus chinensis*, flavonoid metabolites, PCA, OPLS-DA, UPLC-ESI-MS/MS

## Abstract

The goal of this study was to identify and compare the main biomarkers of *Taxillus chinensis* from different hosts. A metabolomics approach utilizing ultra-pressure liquid chromatography coupled with tandem mass spectrometry (UPLC-MS), including cluster analysis, sample correlation analysis and orthogonal partial least squares discriminant analysis, was used to explore the flavonoid metabolites of *Taxillus chinensis* growing on different hosts. Results: The total flavonoids content (up to 30.08 mg/g) in *Taxillus chinensis* from *Morus alba* (CSG) was significantly higher than that from growth on *Liquidambar formosana* (CFG) or *Clausena lansium* (CHG) (*p *< 0.01). There were 23 different metabolites between CSG and CHG, 23 different metabolites between CSG and CFG, and 19 different metabolites between CHG and CFG. The results demonstrated that different hosts exerted a large influence on the metabolites of *Taxillus chinensis*; it was found that CSG differed from CFG and CHG in eleven metabolic compounds, ten of which were upregulated and one of which was downregulated. Most of these metabolites derive from compounds contained in the host plant, white mulberry (*Morus alba*); many feature potent anti-cancer effects. Differences in host can influence the type and abundance of flavonoids in parasitic plants such as *Taxillus chinensis*, which is of great significance to researchers seeking to understand the formation mechanism of *Taxillus chinensis* metabolites. Therefore, attention should be paid to the species of host plant when studying the *Taxillus chinensis* metabolome. Plants grown on *Morus alba* offer the greatest potential for the development of new anti-cancer drugs.

## 1. Introduction

The dried leaf-carrying stems of *Taxillus chinensis* (DC) Danser are commonly used in traditional Chinese medicine. The herb’s functions include dispelling wind-damp, reinforcing the liver and kidney, building strong bones and swollen carbuncles, calming babies in the uterus, etc. It is often used in the treatment of rheumatism and joint pain, hip and knee weakness, weakness of muscles and bones, excessive leakage of meridians, blood leakage during pregnancy, fetal restlessness and dizziness [1].

*T. chinensis* is a semi-parasitic plant with aerial roots that is connected with a host plant, such as the *Morus alba*, through its haustoria. It absorbs water and inorganic salts from the host but can also synthesize carbon compounds through photosynthesis. *T. chinensis* can live on several kinds of host plants, including *Morus alba*, *Clausena lansium*, *Liquidambar formosana*, tea-tree oil, longan and others, but the most common host plant is *Morus alba*. According to the *Classified Materia Medica* [2]: “When you taste the real *T. chinensis* plant, you can experience very good healing effects”. The *Compendium of Materia Medica* [3] claims: “Now there are many *T. chinensis* from other trees on the market to pass off as authentic *T. chinensis*, these herbs have different effects from the real *T. chinensis* and may be harmful to the body.” Physicians of all dynasties attached great importance to the host of *T. chinensis*, and plants growing on *Morus alba* as host were widely recognized by physicians as the best [4]. However, according to the current *Chinese Pharmacopoeia* [1], the herb known as *T. chinensis* contains the dried leaf stems and branches of *Taxillus chinensis* (DC) Danser, and gives no clear restriction on the host of medicinal material of *T. chinensis,* as long as the medicinal material is from *T. chinensis* (DC) Danser and features undetectable cardiac glycosides. The components of the herbal preparation include flavonoids, alkaloids, terpenoids, polypeptides, proteins, lectins, polysaccharides, and organic acids. The main active components are flavonoids [5], such as, quercetin, polygonin and rutin [6], which are considered to be the source of its medicinal effects, such as dispelling wind-damp, reinforcing the liver and kidneys, building strong bones, etc. [7,8,9]. *T. chinensis* flavonoids feature antitumor [10,11], hypotensive, hypoglycemic [12], antioxidant [13], and anti-allergic properties, and are capable of memory enhancement, improving the function of joints [14], reducing inflammation, and stopping pain [15,16,17,18]. Because of differences in hosts, the metabolic activities of the parasitic plant itself are also different, resulting in different chemical components [19]. Relevant research reports have highlighted significant differences in total flavonoid content of *T. chinensis* parasitizing different hosts [20,21,22,23]. The contents of quercetin and quercitrin were also found to differ according to the species of host plant [24]. In spite of this considerable body of research, the metabolites of flavonoids have not been investigated. In this study, an integrated detection system involving ultra-pressure liquid chromatography combined with electrospray ionization–tandem mass spectrometry (UPLC-ESI-MS/MS) coupled with clustering analysis, principal component analysis (PCA), and orthogonal signal correction and partial least squares-discriminant analysis (OPLS-DA) were used to investigate the differences in flavonoid metabolites of *T. chinensis* from *Morus alba*, *Clausena lansium* and *Liquidambar formosana*, to provide a theoretical basis for the medicinal use of *T. chinensis.*

## 2. Results and Analysis

### 2.1. Quantitative Analysis of Total Flavonoid Content in T. chinensis from Different Hosts

**Figure 1:**
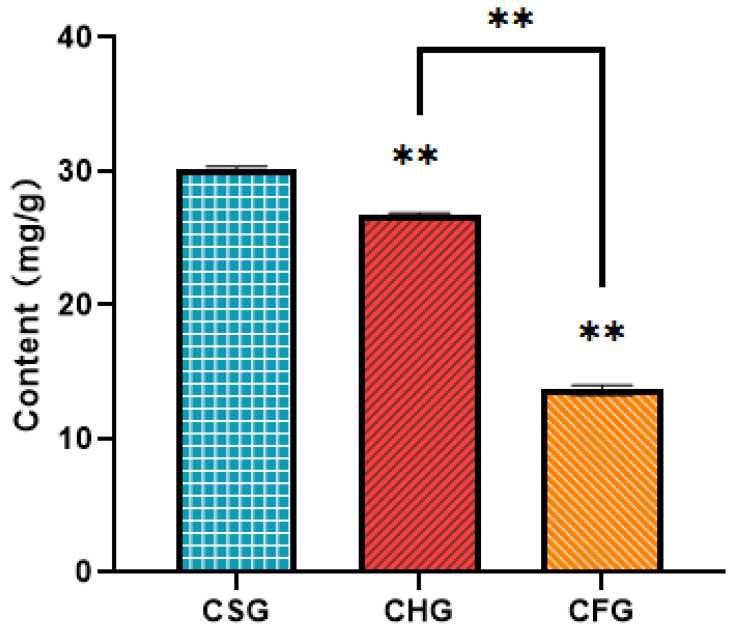
Content analysis of total flavonoids in *T. chinensis* from different hosts (CSG, CHG, and CFG). ** above the histogram indicates statistical significance (*p* < 0.01).

This study selected three different hosts of *T. chinensis* as research subjects. As shown in Figure 1, the total flavonoid content of *T. chinensis* from *Morus alba* (CSG) was significantly higher than that from *Liquidambar formosana* (CFG) and *Clausena lansium* (CHG) (*p* < 0.01), reaching a maximum level of 30.08 mg/g. The total flavonoid content from CHG (26.67 mg/g) (*p* < 0.01) was significantly higher than that from CFG, which featured the lowest value, 13.65 mg/g. These differences in total flavonoid content confirm that it is specifically related to the species of host.

### 2.2. Comprehensive Analysis of Metabolite Composition

Based on the Metware (Wuhan) metabolite database and the relevant mass spectrometry database, a multimodal graph was constructed showing the MRM metabolites detected through the triple quadrupole screening ions of each sample and the characteristics of the ions in the detector signal intensity (CPS). The main metabolites were qualitatively identified and quantitated with MultiQuant software. A total of 125 flavonoid metabolites in 13 categories was identified in *T. chinensis* plants from the three host species. Among these, 117 in 13 categories were found in *T. chinensis* growing on *Morus alba* (CSG). The metabolite composition of *T. chinensis* on *Liquidambar formosana* (CFG) consisted of 100 compounds in 13 categories. *T. chinensis* on *Clausena lansium* (CHG) contained 107 metabolites in 13 categories. Most metabolites were detected and identified in all the samples.

### 2.3. PCA Results

**Figure 2:**
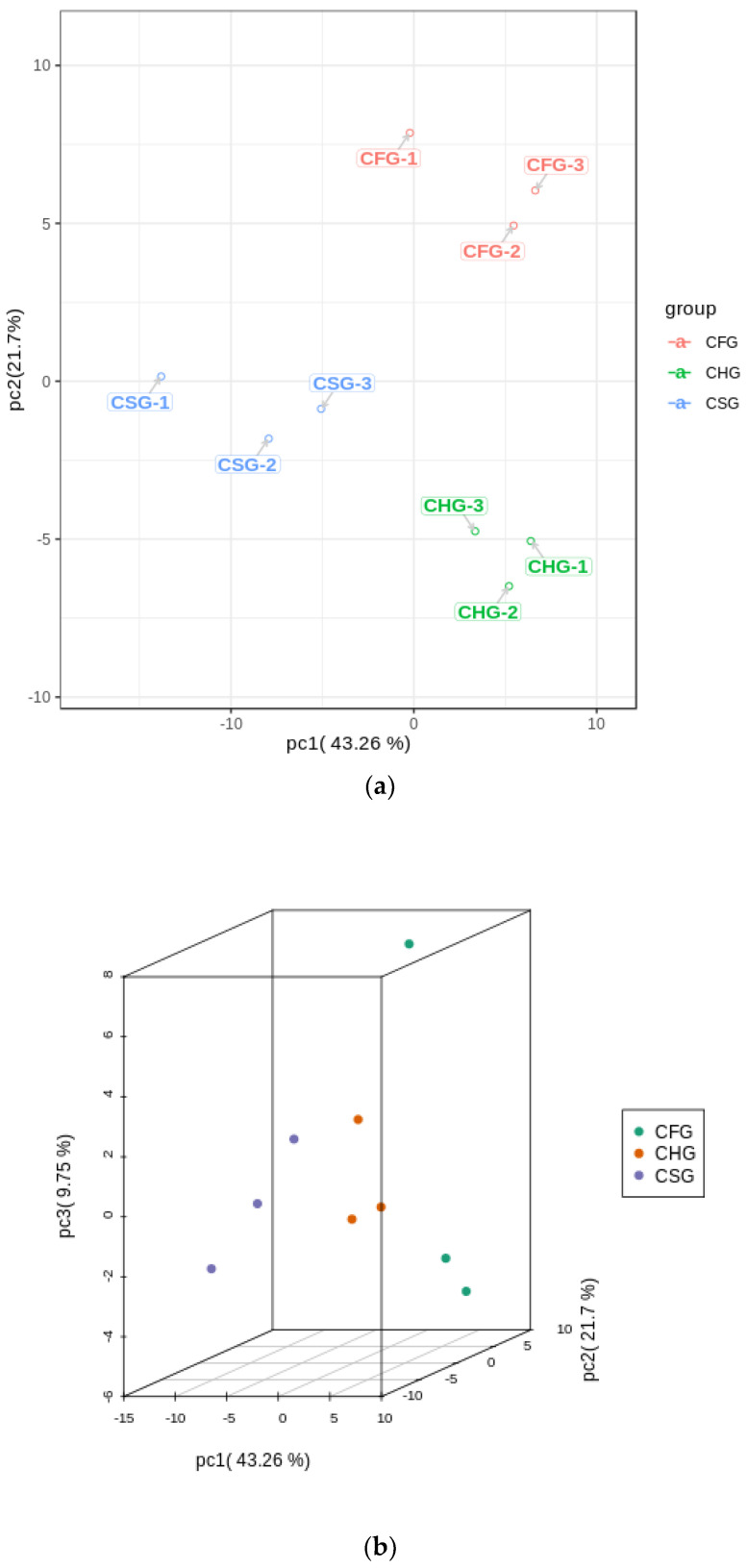
Differential metabolite analysis using principal component analysis (PCA). (**a**) 2D figure; (**b**) 3D figure.

Through a principal component analysis of the samples, the degree of variation between and within the groups of each sample was determined. The analysis resulted in 9 principal components. Principal component 1 (PC1) contributed 43.26% to the variance, while principal component 2 (PC2) accounted for 21.7%. The three groups of samples displayed an clear separation trend in the two-dimensional diagram (Figure 2), and the PCA results could generally reflect the metabolite differences between the three groups of samples.

### 2.4. OPLS-DA Results

**Figure 3:**
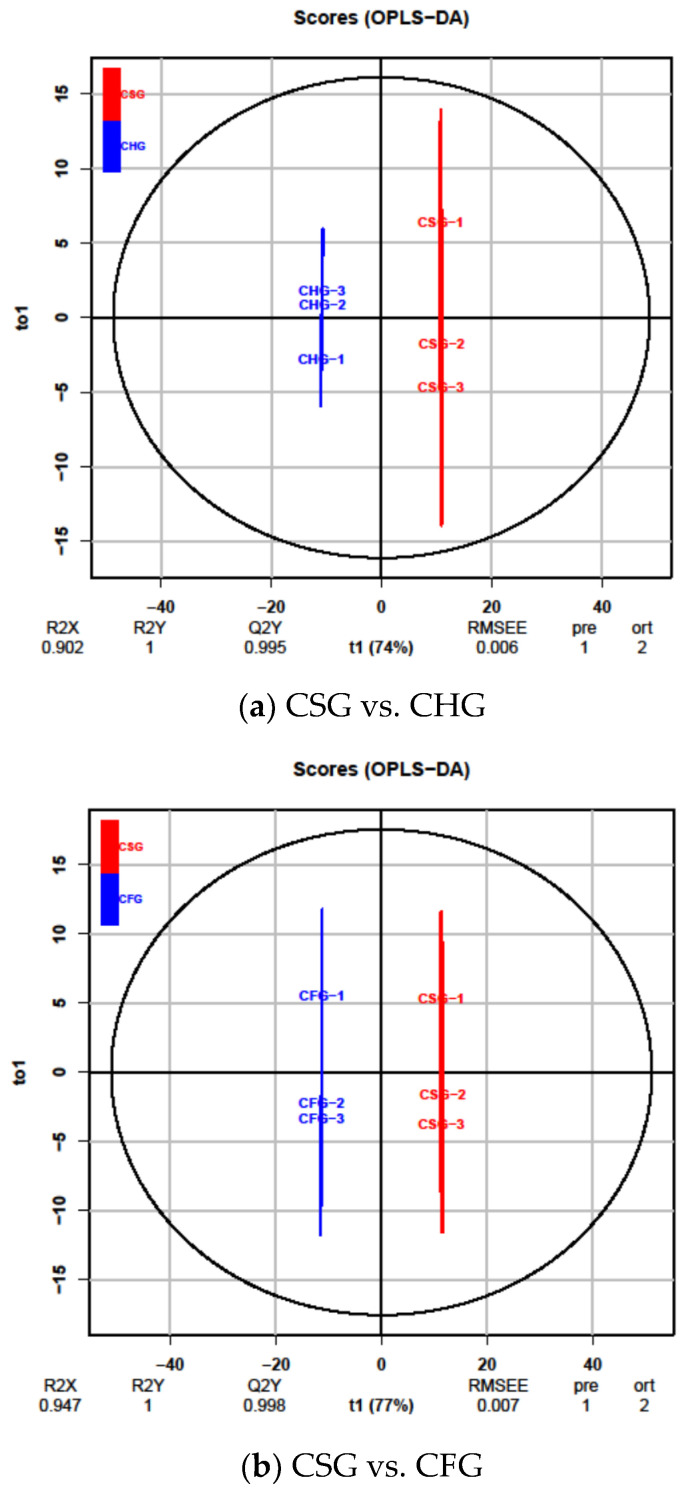

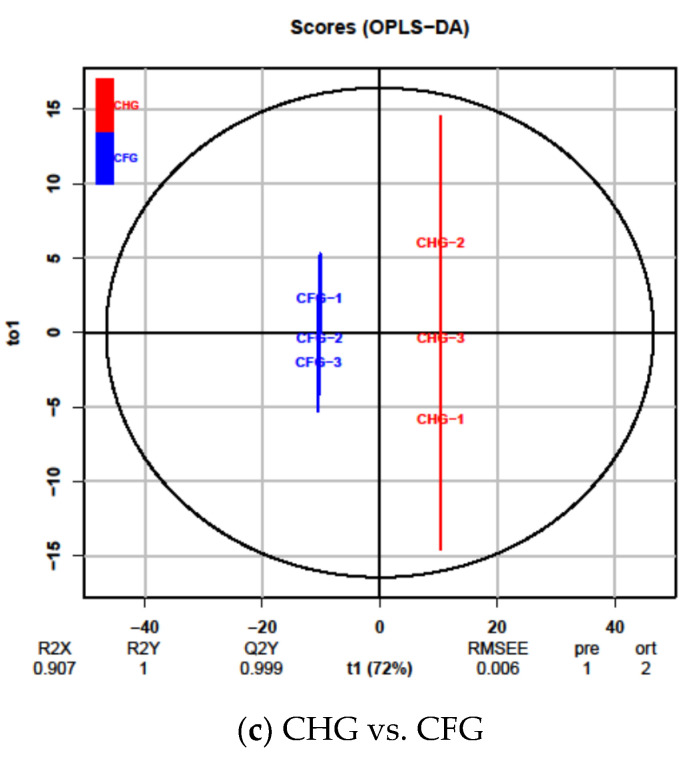
Differential flavonoid metabolite analysis on the basis of orthogonal signal correction and partial least squares discriminant analysis (OPLS-DA); (**a**–**c**) OPLS-DA model plots for comparison of CSG vs. CHG, CSG vs. CFG and CHG vs. CFG.

Although PCA analysis can effectively extract the main information, it is insensitive to variables with low correlation. PLS-DA can maximize the differences between groups, which is conducive to searching for differentially produced metabolites. Orthogonal partial least squares discriminant analysis (OPLS-DA) combines orthogonal signal correction (OSC) and PLS-DA methods to screen differential variables by removing irrelevant differences. According to the OPLS-DA model, the metabolome data of 125 flavonoids were analyzed, and the differences between the samples was rendered significantly obvious. In the comparison between *T. chinensis* from *Morus alba* (CSG) and *T. chinensis* from *Clausena lansium* (CHG), R2X = 0.902, R2Y = 1, and Q2 = 0.995 (a); in the comparison between *T. chinensis* from *Morus alba* (CSG) and *T. chinensis* from *Liquidambar formosana* (CFG), R2X = 0.947, R2Y = 1, Q2 = 0.998 (b); in the comparison of *T. chinensis* from *Clausena lansium* (CHG) to *T. chinensis* from *Liquidambar* *formosana* (CFG), R2X = 0.907, R2Y = 1, Q2 = 0.999 (c), all Q2 > 0.9. The above model was proven to be effective, and better than the PCA model (Figure 3).

### 2.5. Differential Metabolite Identification and Screening

**Figure 4:**
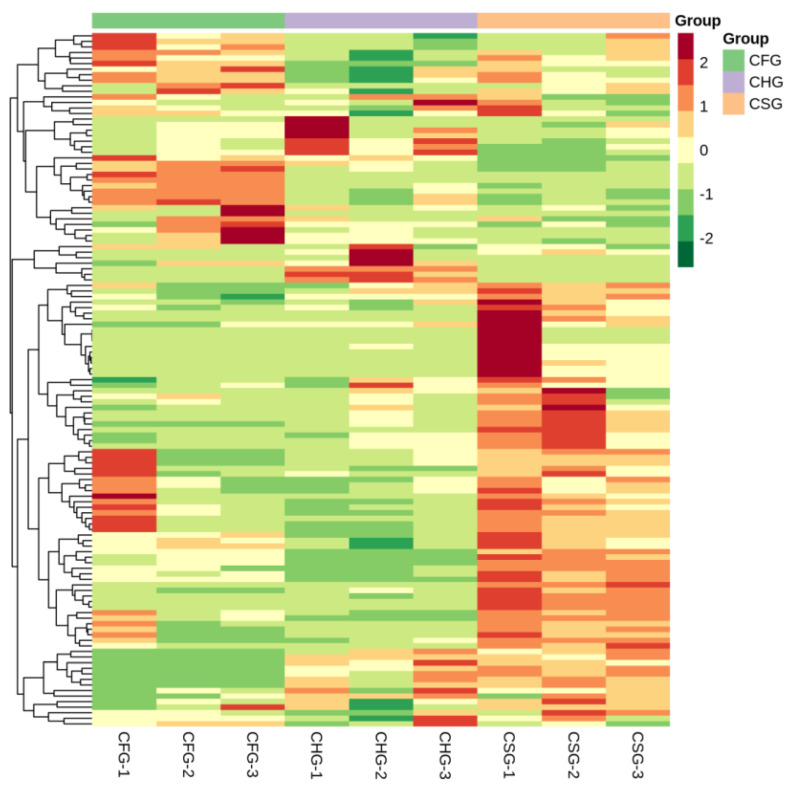
Heatmap of differential metabolites. Metabolite clustering heatmap. The metabolite content data were normalized. Each sample is represented by a column and each metabolite by a row. Red bars indicate high abundance, while green bars indicate low relative abundance (color key scale next to heat map).

In order to facilitate the identification of the change-rule of the metabolites, those with significant differences were normalized by processing and a clustering heatmap was drawn (Figure 4). Figure 4 presents a simple and intuitive depiction of the changes in the metabolites. CSG featured 123 different flavonoid metabolites, CHG 120, and CFG 114, the specific results are shown in Table S1. The content of most of the metabolites in *T. chinensis* from CSG was u-regulated, which was significantly different from what was observed in CHG and CFG.

**Figure 5:**
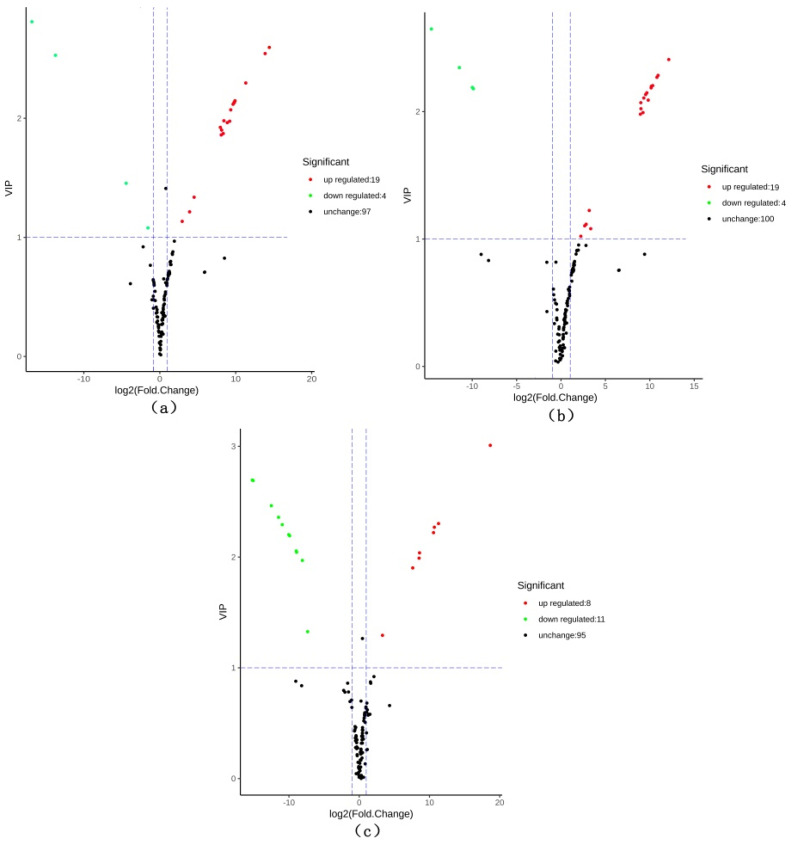
Volcano maps of differential metabolites. (**a**) CSG vs. CHG; (**b**) CSG vs. CFG; and (**c**) CFG vs. CHG. Each dot represents a metabolite; the horizontal axis represents the log of the difference of a metabolite in the two samples, log_2_(fold change). The vertical axis represents the importance of the variable (VIP). The larger the absolute value of the abscissa, the more significant the difference is, which results in screening of the target differential metabolites. The green dots indicate that the differential metabolite was downregulated, the red dots indicate that the differential metabolite was upregulated, and the black dots indicate that the differential metabolite was not significantly changed.

Based on the OPLS-DA results, the variable importance in project (VIP) of the OPLS-DA model was analyzed from the obtained multivariate data. The VIP value represents the intensity of influence of corresponding to metabolite differences between groups in the classification discrimination of each group of samples in the model. It is generally recognized that a metabolite with VIP ≥ 1 is significantly different. The criteria for screening included a fold change value of ≥2 or ≤0.5 and a VIP value of ≥1. Compared with CHG, 23 differential metabolites in CSG were screened, including 19 upregulated and 4 down-regulated metabolites. Compared with CFG, 23 differential metabolites were screened, including 19 upregulated and 4 downregulated metabolites. Comparing CHG with CFG, a total of 19 metabolites were screened, among which 11 metabolites of CHG were upregulated, and 8 metabolites of CFG were upregulated. Figure 5 displays the screening process for the differentials.

**Figure 6:**
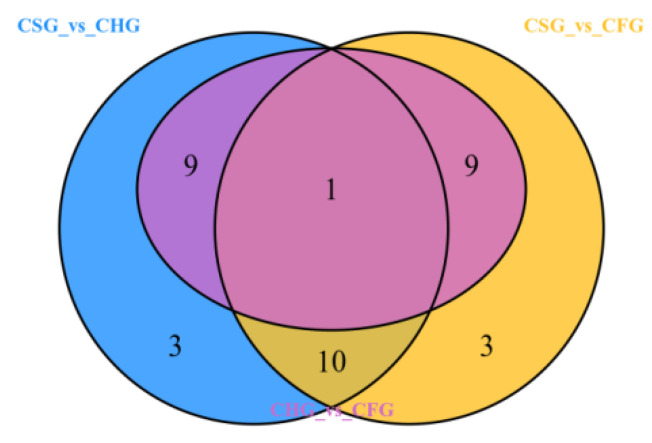
Venn diagram showing numbers of metabolites at intersections.

**Figure 7:**
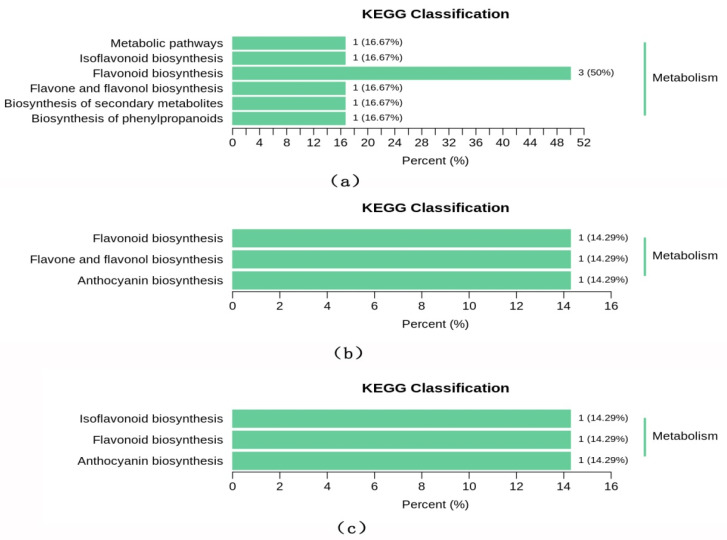
KEGG enrichment of differential metabolites. (**a**–**c**) KEGG classification of the differential metabolites in comparison group CSG versus CHG, CSG versus CFG and CHG versus CFG.

The Venn diagram displays the number of overlapping and unique differential metabolites for each of the group comparisons: CSG vs. CHG, CSG vs. CFG and CHG vs. CFG (Figure 6). The results demonstrate that CSG vs. CHG and CSG vs. CFG intersected 11 different metabolic compounds, indicating that these 11 components were characteristic of CSG where the relative content of 10 metabolites was upregulated (Table 1). There was only one component in the intersection of all the groups, indicating that there were large differences in the differential metabolites of each sample. The KEGG classification results and enrichment analysis (Figure 7a–c) indicate that the differential flavonoid metabolites of the comparison groups were involved in the biosynthesis of flavonoids, flavones, flavonols, anthocyanins and isoflavonoids.

## 3. Discussion

*T. chinensis* is a medicinal herb that parasitizes other plants and uses their nutrients for its growth. In traditional Chinese medicine, *T. chinensis* that grows on *Morus alba*, is considered to offer the best therapeutic efficacy. This variety of the herb can be used as a tea or for the development of high-value drugs. It is commonly known that the composition of parasitic plants such as *T. chinensis* is greatly affected by the type of host plant on which they grow. It is difficult to distinguish the varieties of *T. chinensis* from different hosts by appearance and techniques such as thin layer chromatography must be used for physico-chemical identification. In this study, the effects of different host plants on the total flavonoid content and metabolomics components in *T. chinensis* were studied to clarify the differences between the hosts in their influence on the composition of active medicinal compounds in the parasite. The experiments demonstrated that growth on CSG (*Morus alba*) resulted in *T. chinensis* with a significantly higher content of total flavonoids than CFG (growing on *Liquidambar formosana*) and CHG (growing on *Clausena lansium*). Flavonoids often feature anti-inflammatory properties, enhance immunity, improve joint movement and reduce pain, so it is logical for practitioners of traditional Chinese medicine to use parasitic herbs growing on *Morus alba.* The metabolomics study of the CSG, CFG and CHG samples revealed that principal component analysis (PCA) and orthogonal signal correction and partial least squares discriminant analysis (OPLS-DA) demonstrated that the three samples were clearly differentiated. Although the three samples were the of the same basic medicinal material, different hosts exerted a significant effect on the metabolites of the medicinal material, which was confirmed by the heatmap. The enrichment analysis of the differential metabolites revealed that they were mainly involved in the biosynthesis of flavonoids, flavones, flavonols, anthocyanin, and isoflavonoids. By comparing the differential metabolites of CSG vs. CHG and CSG vs. CFG, we found 11 unique differential metabolites in CSG, namely luteolin-7,3′-di-O-beta-d-glucoside, pinocembrin (dihydrochrysin), nigrasin A, artonin E2, kuwanon S2, 8-C-hexosyl-luteolin O-hexoside, sanggenon F/H, anggenon M, kuwanon D, sanggenol L and luteolin 7-O-glucoside (cynaroside).

Pinocembrin is a natural flavonoid compound that exerts anti-inflammatory and antioxidant effects, but can also be used as a drug to treat allergy [25] and as a neuroprotectant in cerebral ischemia injury [26]. It has also been reported that pinocembrin has inhibitory effects on lung cancer cells [27]. Artonin is a type of prenylated flavonoid that has been found to have potent inhibitory effects against triple negative breast cancer cells and epidermoid carcinoma A431 cells [28,29]. Its pharmacological activity is similar to that of artonin E2, which showed strong inhibitory activity against ovarian cancer cell line 1A9 [30,31]. Sanggenone D can induce tumor cells to differentiate into normal cells, induce the apoptosis of hepatocellular carcinoma cells, and inhibit the proliferation of B16F0 cells, B16F10 cells, F10 cells and HepG2 cells [32]. Sanggenol L is a flavonol compound that is effective as a chemotherapeutic agent in the treatment of various cancers, such as ovarian and melanoma [33,34]. The content of pinocembrin, artonin E, sanggenone D and sanggenol L metabolic components in CSG was significantly higher than in CFG and CHG. These components can induce apoptosis and exhibit strong inhibitory activity against cancer cells, indicating that CSG offers potential for development into an effective anticancer drug [35,36]. However, compared to CHG and CFG, the content level of luteolin in CSG was significantly downregulated. An analysis of these 11 differential metabolites demonstrated that artonin E2, sanggenon F/H, kuwanon S2, sanggenon M, kuwanon D and sanggenol L were all enriched components of *Morus alba* branches and roots, indicating that *T. chinensis* absorbed these compounds by parasitizing *Morus alba*, thereby changing its own metabolic components. Similarly, the analysis of the CFG and CHG samples detected almost none of these compounds. It is clear that different hosts exert great influence on the metabolite content of *T. chinensis*.

## 4. Plant Materials and Treatments

### 4.1. Plant Materials

*Taxillus chinensis* (DC) Danser were collected from *Morus alba* L, *Liquidambar formosana* Hance, and *Clausena lansium* (Lour.) in Anping Town, Cenxi County, Wuzhou City, Guangxi. The habitats of the collected herbs were all similar. Three replicates of each sample were taken with a centrifuge tube (50 mL) and then quickly frozen in liquid nitrogen and stored at −80 °C until use.

### 4.2. Metabolite Extraction

The three groups of samples, stored at −80 °C, were freeze-dried in vacuo, and then stems and leaves were mixed at a ratio of 1:2 and ground (30 Hz, 1.5 min) to powder using a grinding machine (Shanghai, China, MM 400, Retsch). Samples for extraction (100 mg) were dissolved in 1.0 mL of 70% methanol and kept at 4 °C overnight, during which time they were vortexed three times to improve the extraction rate. After extraction, the samples were centrifuged at 10,000× *g* the supernatants were carefully removed, and then filtered through a 0.22 μm microporous membrane for LC-MS/MS analysis.

### 4.3. Calibration Curves

Different qualities of rutin were transferred into 25 mL volumetric flasks and 2 mL of aluminum chloride (AlCl_3_, 0.1 mol/L) and 1 mL of sodium acetate (CH₃COONa, 0.1 mol/L) were added [37]. The volume was up to 25 mL with 65% ethanol. The flasks were shaken well, and the color was allowed to develop for 20 min. The blank solution was aluminum chloride, sodium acetate and 65% ethanol and the sample absorbance was determined at 407 nm. The standard curve was drawn using rutin concentration in mg/mL as the abscissa and A as the ordinate. Standard curve range: 0.012–0.032 mg/mL.

### 4.4. Determination of Total Flavonoid Content

A pipet extract of the total flavonoids from *T. chinensis* was taken and the color was developed using the ‘4.3’ method. Its absorbance was measured at 407 nm in UV and the total flavonoid content was calculated according to the following formula:

Total flavonoid content=total flavonoids extract (mg)÷ sample quantity (g)

### 4.5. LC-MS/MS Analysis of Metabolites

The samples were isolated by UPLC (Shanghai, China, Shim-pack UFLC Shimadzu CBM30A) and identified by tandem mass spectrometry (MS/MS) (Shanghai, China, Applied Biosystems 6500 QTRAP). The LC separations were performed using a Waters Acquity UPLC HSS T3 C18 chromatographic column (Shanghai, China, 1.8 μm, 2.1 mm × 100 mm). The mobile phase included 0.04% acetic acid in ultra-pure water and 0.04% acetic acid in acetonitrile. The elution gradient was as follows: water/acetonitrile (95:5, *v*/*v*) at 0 min, 5:95 (*v*/*v*) at 11.0 min, 5:95 (*v*/*v*) at 12.0 min, 95:5 (*v*/*v*) at 12.1 min, and 95:5 (*v*/*v*) at 15.0 min. The sample size was 5 μL, the flow rate was 0.4 mL/min and the column temperature was 40 °C. The effluent was alternately connected to the electrospray ion source (ESI) of the triple quadrupole (QQQ) mass spectrometer. The mass spectrometry conditions were: ESI temperature 550 °C, voltage 5500 V and curtain gas (CUR) 25 psi.

### 4.6. Quality Control

**Figure 8:**
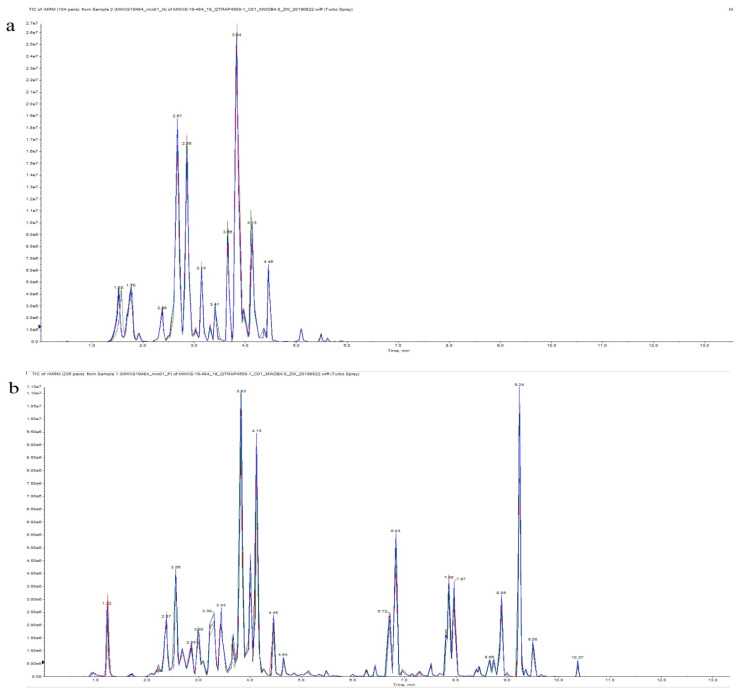
Mass spectrometry quality control (QC) total ion current (TIC) superposition diagram. (**a**) Diagram of TIC of negative-ion multiple reaction monitoring (MRM). (**b**) TIC of positive-ion MRM diagram.

Quality control (QC) equivalent samples of the *T. chinensis* extract from the three different host groups were prepared in triplicate using the same sample processing and testing methods. For the QC testing process, a QC sample was inserted into every ten experimental samples during the analytical run to determine the repeatability of the process. Overlapping displays for analysis of the total ion current (TIC) were drawn as maps for the mass spectrometry analysis of different quality control samples (QC). The total ion flow map of the QCs is presented in Figure 8.

### 4.7. Metabolite Identification and Analysis

The compounds were identified through a comparison with known spectral data in MS and MS/MS of the MVDB V2.0 database and with the self-built database MWDB (Metware Biotechnology Co., Ltd. Wuhan, China). The structural analysis of the metabolites was performed using MassBank (http://www.massbank.jp/), METLIN (http://metlin.scripps.edu/index.php) and MDB (http://www.hmdb.ca/) (The access date for the three databases is 30 October 2019).

The metabolite quantification was completed by triple quadrupole mass spectrometry multiple reaction monitoring (MRM). To obtain the metabolic substance spectra of the different samples, the area of the mass spectrum peak was integrated and the metabolite peaks in different samples were corrected [38]. In MRM mode, the quadrupole mass first screened the parent ions (Q_1_) of the target substance and excluded the corresponding ions of other molecular weight substances to eliminate the interference initially. The parent ion was ionized in an ion collision chamber and fractured to form many fragments. The fragment ions were filtered out through the triple quadrupole mass to eliminate the interference of non-target ions and obtain a characteristic fragment ion (Q_3_), so that the quantification was more accurate and the repeatability was better. Next, the peak area integration was carried out for the mass spectrum peaks of all the substances and the software Analyst 1.6.3 (Waltham, MA, USA) was used to process the mass spectrum data. Multivariate statistical analysis was used to perform a principal component analysis (PCA) and a cluster analysis on the three groups of samples; the stability and reliability of the model were predicted by using partial least squares discriminant analysis (PLS-DA) and orthogonal partial least squares discriminant analysis (OPLS-DA). Differential metabolites were screened for multi-dimensional statistical VIP values. The criteria for screening included a fold change value of ≥2 or ≤0.5 and a VIP value of ≥1. A univariate analysis of the *p* values and difference multiples was performed, followed by a clustering analysis; heatmaps were constructed using the PHEATMAP program in R software (V3.3.2, Auckland, New Zealand) to analyze the different metabolic components of the three selected hosts of *T. chinensis* parasitism. Hierarchical clustering was performed for the different metabolites in each group, and the corresponding metabolites obtained were submitted to the KEGG website for correlation pathway analysis.

## 5. Conclusions

In this paper, the flavonoid metabolites in *T. chinensis* growing on three different host plants (*Morus alba*, *Clausena lansium* and *Liquidambar formosana*) were systematically analyzed and identified by LC-MS/MS using a widely targeted metabolome. For comparison with the parasitic medicinal herbs of *Clausena lansium* host plants, 23 differential metabolites in parasitic medicinal herbs of *Morus* *alba* plants were screened, including 19 upregulated metabolites and 4 downregulated metabolites. For comparison with parasitic medicinal herbs of *Liquidambar formosana* plants, 23 differential metabolites were screened, including 19 upregulated metabolites and 4 downregulated metabolites. The composition of the *T. chinensis* medicinal materials were clearly influenced by the host plant. Medicinal herbs that use *Morus alba* as the host, such as *T. chinensis*, frequently feature a metabolite profile and composition with strong anti-cancer, anti-inflammatory, and analgesic activities, good for improving joint health and other pharmacological actions. Because of these beneficial health properties, traditional Chinese medicine practitioners are more likely to make use of medicinal materials from *Morus alba*-hosted *T. chinensis*.

## Figures and Tables

**Table 1:** List of biologically active, upregulated flavonoid metabolites detected in CSG.

Index	Rt (min)	Q_1_	Q_3_	Molecular Weight	Ionization Model	Formula	Compounds
pmp000595	3.80	611.16	287.05	610.13	[M+H]^+^	C_27_H_30_O_16_	Luteolin-7,3′-Di-O-beta-d-glucoside
mws0789	6.00	255.10	107.01	256.06	[M−H]^−^	C_15_H_12_O_4_	Pinocembrin (dihydrochrysin)
pmp000836	7.40	455.20	379.12	454.14	[M+H]^+^	C_25_H_26_O_8_	Nigrasins A
pmp000821	8.00	421.20	309.04	420.14	[M+H]^+^	C_25_H_24_O_6_	Artonin E2
pmp000815	8.60	407.20	165.02	406.16	[M+H]^+^	C_25_H_26_O_5_	Kuwanon S2
pmb0663	4.20	611.10	383.08	610.15	[M+H]^+^	C_27_H_30_O_16_	8-C-hexosyl-luteolin O-hexoside
pmp000809	6.00	355.10	229.05	354.10	[M+H]^+^	C_20_H_18_O_6_	Sanggenon F/H
pmp000829	6.70	437.20	363.09	436.13	[M+H]^+^	C_25_H_24_O_7_	Sanggenon M
pmp000825	8.00	423.20	153.02	422.15	[M+H]^+^	C_25_H_26_O_6_	Kuwanon D
pmp000822	7.00	423.20	311.07	422.15	[M+H]^+^	C_25_H_26_O_6_	Sanggenol L

## Data Availability

Not applicable.

## Supplementary Materials

The following are available online. Table S1: List of flavonoid metabolites in three parasitic plants.

## Sample Availability

Samples of the compounds are *Taxillus chinensis* on different hosts available from the authors.
